# Correction: Development of highly active anti-*Pneumocystis* bisbenzamidines: insight into the influence of selected substituents on the *in vitro* activity

**DOI:** 10.1039/c7md90042b

**Published:** 2017-11-07

**Authors:** D. Maciejewska, J. Żabiński, M. Rezler, P. Kaźmierczak, M. S. Collins, L. Ficker, M. T. Cushion

**Affiliations:** a Department of Organic Chemistry , Faculty of Pharmacy , Medical University of Warsaw , Banacha 1 Street , 02-097 Warsaw , Poland . Email: dmaciejewska@wum.edu.pl; b Division of Infectious Diseases , Department of Internal Medicine , University of Cincinnati College of Medicine , 231 Albert Sabin Way , Cincinnati , OH 45267 , USA . Email: cushionmt@ucmail.uc.edu; c Cincinnati Veterans Affairs Medical Center , 3200 Vine Street , Cincinnati , OH 45220 , USA

## Abstract

Correction for ‘Development of highly active anti-*Pneumocystis* bisbenzamidines: insight into the influence of selected substituents on the *in vitro* activity’ by D. Maciejewska *et al.*, *Med. Chem. Commun.*, 2017, **8**, 2003–2011.



## 

The authors regret that on page 7 of their article the name for compound **13** contains an error. The correct name for **13** is: 1,5-bis(4-amidino-2-nitrophenoxy)-3-oxapentane dihydrochloride (with -2- instead of -3-nitrophenoxy).

The Royal Society of Chemistry apologises for these errors and any consequent inconvenience to authors and readers.

